# Alterations of the *WNT7A* Gene in Clear Cell Renal Cell Carcinomas

**DOI:** 10.1371/journal.pone.0047012

**Published:** 2012-10-08

**Authors:** Aleksandr G. Kondratov, Sergiy M. Kvasha, Liubov A. Stoliar, Alina M. Romanenko, Yury M. Zgonnyk, Vasily V. Gordiyuk, Elena V. Kashuba, Alla V. Rynditch, Eugene R. Zabarovsky, Vladimir I. Kashuba

**Affiliations:** 1 Department of Molecular Oncogenetics, Institute of Molecular Biology and Genetics, National Academy of Science, Kyiv, Ukraine; 2 Institute of Urology, Academy of Medical Sciences, Kyiv, Ukraine; 3 Department of Functional Genomics, Institute of Molecular Biology and Genetics, National Academy of Science, Kyiv, Ukraine; 4 Department of Microbiology, Tumor and Cell Biology (MTC), Karolinska Institute, Stockholm, Sweden; 5 R.E. Kavetsky Institute of Experimental Pathology, Oncology and Radiobiology, National Academy of Science, Kyiv, Ukraine; 6 Laboratory of Structural and Functional Genomics, Engelhardt Institute of Molecular Biology, RAN, Moscow, Russia; 7 Department of Clinical and Experimental Medicine, Faculty of Health Sciences, Linkoping University, Linkoping, Sweden; Institut national de la santé et de la recherche médicale, France

## Abstract

*WNT7A* (wingless-type MMTV integration site family, member 7A) is a known tumor suppressor gene of non-small cell lung carcinomas (NSCLC) and is frequently inactivated due to CpG-island hypermethylation in human cancers. The members of WNT family are involved in cell signaling and play crucial roles in cancer development. In the present work hypermethylation of the *WNT7A* gene was detected in 66% (29/44) of analyzed clear cell renal cell carcinomas (RCCs) using methyl-specific PCR (MSP). Moreover, bisulfite sequencing confirmed intensive hypermethylation of the 5′-CpG island of the *WNT7A* gene. Methylation analysis revealed positive correlations between tumor stage, Fuhrman nuclear grade and *WNT7A* hypermethylation. Additionally, restoration of *WNT7A* gene expression in the A498 cell line by 5-aza-2′-deoxycytidine treatment confirmed a direct contribution of hypermethylation in silencing of the *WNT7A* gene. High frequency of loss of heterozygosity (LOH) was demonstrated on chromosome 3p25 in regions surrounding the *WNT7A* gene. The frequent down-regulation of *WNT7A* gene expression was detected in 88% (15/17) of clear cell RCCs. We have also shown that the *WNT7A* gene possesses tumor suppression function by colony-formation and cell proliferation assays in RCC cell lines. In summary, the *WNT7A* gene is inactivated by genetic/epigenetic alterations in clear cell RCC and demonstrates tumor suppressor properties.

## Introduction

Renal cell carcinoma (RCC) is the most common type of kidney cancer, responsible for 3% of human malignancies [1]. Clear cell RCC accounts for 70–75% of RCC and is distinguished by a set of genetic and epigenetic abnormalities [2]. It is known that inactivation of tumor suppressor genes is a frequent event for sporadic clear cell RCCs. DNA methylation and deletions are the most common mechanisms of inactivation of tumor suppressor genes in clear cell RCCs [3]–[6]. Moreover, it was shown that abnormalities of human chromosome 3 significantly contributed to clear cell RCCs development. Arai et al. identified chromosome 3 as one of the most affected by genetic/epigenetic alterations in clear cell RCCs [7], [8]. In particular, DNA methylation of promoter regions was shown for *RASSF1*, *FHIT*, *LRRC3B*, *VHL* and other well-characterized tumor suppressor genes in clear cell RCCs [9]–[12].

In previous work we have found that *WNT7A* associated locus is subjected to genetic/epigenetic alterations in set of RCC’s using NotI-microarray analysis [13]. The NotI-microarray technology allows to search for genetic (deletion, amplification) and epigenetic (DNA methylation) alterations of genes/loci simultaneously, due to the fact that NotI sites are frequently associated with promoter regions of genes [14]. This technology was used to search for such potential tumor suppressor genes like *LRRC3B* [15], [16], *Fibulin3* [17], *RBSP3* [18] and other genes [19].

*WNT7A* is a known tumor suppressor gene of non-small cell lung carcinomas (NSCLC) [20]–[22] and is frequently inactivated due to CpG-island hypermethylation in such human cancers as lung [19], [23], [24], pancreatic [25] and oral squamous cell carcinomas (OSCC) [26].

The members of the WNT family are involved in cell signaling through canonical [27] (β-catenin dependent) and non-canonical pathways such as Planar Cell Polarity [28] or Wnt/Calcium [29] (β-catenin independent). In the canonical pathway, interaction of WNT proteins with the Frizzle cell membrane receptor results in inhibition of glycogen synthase kinase 3 activity that acts as a negative regulator of β-catenin accumulation. Inhibition of glycogen synthase kinase 3 prevents proteasome-mediated degradation of β-catenin that results in cytoplasmic accumulation of β-catenin with subsequent translocation to the nucleus. The nuclear portion of β-catenin binds to the TCF/LEF family of transcription factors and induces transcription of target genes [30]. Noteworthy, the important role of WNT signaling in the mesenchymal-epithelial transition of metanephric progenitors and in the terminal epithelial differentiation during the kidney development was assumed [31], [32].

At present, the role of the WNT genes in carcinogenesis is rather controversial because several members such as *WNT2* were shown to possess oncogenic features [33], while other members such as *WNT5A* were reported to act as tumor suppressors [34]. The behavior of the *WNT7A* gene in human cancer is tissue-specific. In lung cancer and leukemias *WNT7A* was characterized as a tumor suppressor gene [20]–[22], [35]. Additionally, it was shown that inactivation of *WNT7A* through DNA hypermethylation stabilizes the cancer phenotype of OSCC cell lines [26]. However, the *WNT7A* gene has oncogenic properties in ovarian cancer [36], [37].

In the present study we determined the genetic and epigenetic alterations of the *WNT7A* gene in clear cell RCCs. A correlation exists between genetic/epigenetic alterations and down-regulation of *WNT7A* gene expression. In addition, re-expression of the *WNT7A* gene in RCC cell lines inhibits colony formation and cell proliferation.

## Materials and Methods

### Ethics Statements

All patients gave written informed consent. The samples were collected in accordance with the Declaration of Helsinki and approved by the guidelines issued by the Ethic Committee of the Institute of Urology of the Academy of Medical Sciences, Kyiv, Ukraine.

### Total RNA and Genomic DNA Isolation

Forty four tumor samples of clear cell RCCs with 32 non-malignant adjacent normal tissues were obtained from the Institute of Urology of the Academy of Medical Sciences, Kyiv, Ukraine (Table 1). The classification of the tumors based on the staging system of the American Joint Committee on Cancer (TNM) was used [38]. Genomic DNA was purified according to the protocol from Sambrook et al [39]. Total RNA was isolated with the RNeasy Mini Kit (Qiagen, Valencia, CA) following the manufacturer’s recommendations. The quality of the isolated RNA was assessed by electrophoresis.

**Table 1:** Clinical-pathological characteristics of clear cell RCC samples.

Parameters			Means
Age (years)			55 (22–78)
Sex (M/F)			27/17
**Fuhrman nuclear grade**			
Grade 1			11
Grade 2			18
Grade 3			9
Grade 4			6
**Tumor stage**			
Stage I	(T_1_N_0_M_0_)		3
Stage II	(T_2_N_0_M_0_)		29
Stage III		(T_3_N_0_M_0_)	9
		(T_3_N_1_M_0_)	2
Stage IV		(T_3_N_0_M_2_)	1

### Cell Lines Culturing

Human RCC cell lines A498 and KRC/Y were obtained from Bank of cell lines of R. E. Kavetsky Institute of Experimental Pathology, Oncology and Radiobiology, National Academy of Science, (Kyiv, Ukraine) and Karolinska Institute (Stockholm, Sweden), respectively. A498 and KRC/Y cell lines were described earlier in our works [40], [41]. Cell lines A498 and KRC/Y were cultured in RPMI (Sigma-Aldrich, St. Louis, MO, USA) and IMDM media (Life Technology, Carlsbad, CA, USA), respectively. Media were supplemented with 10% fetal bovine serum and penicillin/streptomycin. Transfection of cell lines was performed using Lipofectamine 2000 (Life Technology), according to the manufacturer’s recommendations.

### Methyl-specific PCR (MSP)

Bisulfite treatment of genomic DNA was performed with the EZ DNA Methylation kit (ZYMO Research, Orange, CA, USA) according to manufacturer’s protocol. Modified DNA (50 ng) was used for each PCR with primers described previously [25]: *WNT7A* M-F 5′-GTAGTTCGGCGTCGTTTTAC-3′, *WNT7A* M-R 5′-CGAAACCGTCTATCGATACG-3′, *WNT7A*, U-F 5′-TAGTTTGGTGTTGTTTTATGTTG-3′, *WNT7A* U-R 5′-CCCCAAAACCATCTATCAATAC-3′. PCRs were performed in the following conditions: 95°C - 4 min, then 35 cycles at 95°C - 15 sec, 59–62°C - 20 sec and 72°C - 30 sec, and the final extension at 72°C for 7 min. The PCR products were analyzed by electrophoresis in 10% polyacrylamide gels with subsequent ethidium bromide staining. M.SssI (NEB, Ipswich, MA, USA) methyltransferase-treated and untreated normal DNA was used as positive control in amplification with primers against methylated and unmethylated sequences, correspondingly. To verify the accuracy of the MSP, the PCR products were recovered from agarose gels, cloned in the pJET1.2-vector (Thermo Scientific, Fermentas) and sequenced. Four to five clones were sequenced for each sample.

### Bisulfite Sequencing

Primers for bisulfite sequencing of the CpG-island of the *WNT7A* gene were designed around the MSP primers in the region +8 bp to +356 bp from the transcription start site (NC_000003.11, from 13921263 bp to 13921611 bp): *WNT7A-*BS For 5′-GGGGGTTGGAGGTAGTAG-3′ and *WNT7A-*BS Rev 5′-TTGTTTGGGTTATTTTTTTTTTAGTTTGGGT-3′. The PCR was carried out using 100 ng of bisulfite-treated DNA and the 1xSYBR Green Mix (Thermo Scientific, Fermentas) in the following conditions: 95°C - 10 min, then 40 cycles at 95°C - 15 sec, 58°C - 20 sec and 72°C - 60 sec, and the final extension at 72°C for 7 min. The PCR products were recovered from agarose gels, cloned in the pGEM-T easy vector (Promega, Madison, WI, USA) and sequenced. Around 8 clones were sequenced for each sample.

### Quantitative Reverse Transcriptase PCR (qRT-PCR)

qRT-PCR was used to assess the change of *WNT7A* gene expression in tissue samples and RCC cell lines. Briefly, 2 µg of total RNA were treated with DNAse (Thermo Scientific, Fermentas) and transcribed into cDNA with an Oligo(dT)-primer using the First Strand cDNA Synthesis Kit (Thermo Scientific, Fermentas). qRT-PCR was performed at IQ5 real-time PCR detection system (BioRad, Hercules, CA, USA) at the following reaction conditions: 95°C - 10 min, than 40 cycles at 95°C - 15 sec, 62°C - 20 sec and 72°C - 30 sec. The *TBP* gene served as a reference gene. qRT-PCR was carried out with the previously reported primers [42]. Changes in *WNT7A* gene expression were calculated by the ΔΔCt method using the efficiency coefficient calculated according to Spiess et al [43].

### Loss of Heterozygosity (LOH) Analysis

Detection of LOH of the microsatellite markers D3S2385, D3S1252, D3S2403 was carried out by amplification of the genomic DNA with Cy5-labeled primers and subsequent analysis by automated laser fluorescence system (Pharmacia Biotech, Uppsala, Sweden) [44]–[46]. The amplification reaction was performed using 1 U of DreamTaq (Thermo Scientific, Fermentas) in the following reaction conditions: 95°C - 4 min, 28 cycles at 95°C - 15 sec, 56–58°C - 20 sec, 72°C - 30 sec, and finally 72°C for 7 min. The fluorescence data were processed by Fragment Manager program (Pharmacia). Differences in peak intensity of alleles was calculated by two methods using the height and area of peaks [46], [47]. Decreased ratio of tumor allele intensity compared with normal and ratio less than 70% was accepted as a criterion for presence of LOH simultaneously for both methods of calculation of peak intensity [48]. Primer sequences were taken from the NCBI UniSTS database with the following accession numbers: D3S2385 - G08224, D3S1252 - L02085, D3S2403 - G08301.

### Restoration of *WNT7A* Gene Expression by 5-aza-2′-deoxycytidine Treatment in the A498 Renal Cell Carcinoma Cell Line

For this purpose the A498 cells were treated with 5 µM 5-aza-2′-deoxycytidine (Sigma-Aldrich) for 5 days. A498 cells treated by solvent for 5-aza-2′-deoxycytidine was used as mock control. The medium was replaced daily. After the treatment, total RNA and genomic DNA were isolated. To assess the effect of drug treatment of the A498 cells on the expression and methylation status of the *WNT7A* gene, qRT-PCR and MSP were used as mentioned above. MSP was carried out with the equal amount of bisulfite treated DNA obtained from 5-aza-2′-deoxycytidine and mock treated A498 cells. To detect expression of *WNT7A* and *TBP* genes, qRT-PCR was carried out for 30 and 24 cycles respectively. Level of the *TBP* expression was used as an internal control.

### Colony Formation and Cell Proliferation Tests

For colony formation tests, A498 and KRC/Y cells were transfected with pcDNA3.1-WNT7A and pcDNA3.1-empty vectors. The level of *WNT7A* expression in cell lines after transfection by pcDNA3.1-WNT7A and pcDNA3.1-empty vectors was assessed by qRT-PCR as mentioned above. Cells (40,000-50,000 cells per well) were seeded in 6-well plates the day following transfection in triplicates. Selection on the 400µg/mL of G418 (Sigma-Aldrich) was started 48 h after transfection. Cells were stained by crystal violet after 2 weeks of G418 selection and number of colonies was counted. The experiment was performed in triplicate.

To perform cell proliferation tests, 1000–1500 cells per well were seeded in 96-well plates 24 h after transfection. The number of cells was counted using the Cell Quantification kit (CCK-8) (Sigma-Aldrich) at 0 h, 24 h, 48 h, 72 h and 96 h after plating according to the manufacturer’s recommendations. During cell proliferation tests cells were grown in medium without G418.

### Statistical Analysis

Statistical analysis was performed using STATISTICA 7.0 program (StatSoft Inc, Tulsa, OK, USA). The expression level for different stages of tumors was compared with the t-test for independent groups. The value p<0.05 was considered as a statistically significant difference. The nonparametric Mann-Whitney U Test was used to calculate difference between samples with the methylation or LOH status and the clinical-pathological characteristics. The difference was considered as significant if p<0.05. The Spearman’s rank correlation coefficient was used to calculate correlation between decrease of the gene expression and the hypermethylation/LOH status. The value r_s_ which corresponds to the value p<0.05 was considered as a statistically significant correlation.

## Results

### Determination of *WNT7A* Methylation Status in Clear Cell RCC and Restoration of *WNT7A* Gene Expression after 5-aza-2′-deoxycytidine Treatment

**Figure 1:**
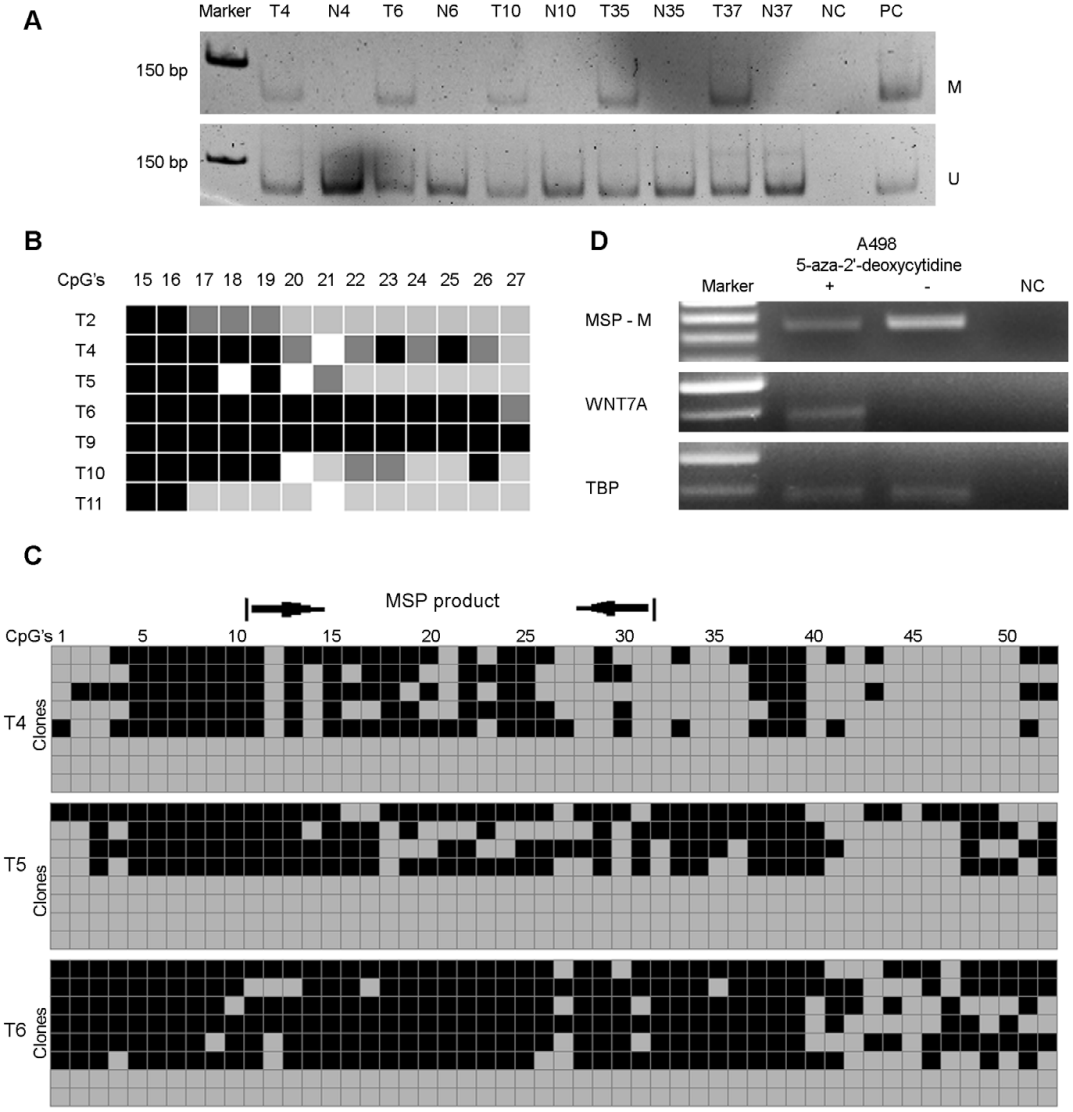
Study of *WNT7A* gene methylation status in clear cell RCC. (A). Representative MSP analysis of the *WNT7A* gene by using methylated (M) and unmethylated (U) specific primers, PC – positive control, M.Sssi treated or untreated normal DNA, NC – negative control (H_2_0), T4, T6, T10, T35, T37: tumor samples, N4, N6, N10, N35, N37: normal samples. (B). Sequencing of MSP products; white squares - 0–19% methylation at the CpG dinucleotide, grey squares - 20–59% methylation at the CpG dinucleotide, dark grey squares - 60–79% methylation at the CpG dinucleotide, black squares - 80–100% methylation at the CpG dinucleotide, T2, T4, T5, T6, T9, T10, T11: tumor samples. (C). Methylation status of the fifty two CpG dinucleotides of the *WNT7A* 5′-CpG island in tumor samples with a methylated *WNT7A* gene, where each CpG dinucleotide is shown by either a black square when methylated or a grey square when unmethylated; arrows indicate position of MSP primers, T4, T5, T6 are tumor samples. (D). Restoration of *WNT7A* expression by 5-aza-2′-deoxycytidine treatment of the A498 cell line, MSP-M - methylation analysis of the *WNT7A* gene by using methylated specific primers, NC – negative control (H_2_0).

To examine the presence of epigenetic alterations of the *WNT7A* gene in clear cell RCCs, the methylation status of the 5′-CpG island of the *WNT7A* gene was first assessed by MSP. The methylation status of the *WNT7A* gene promoter was examined in 44 clear cell RCCs and 28 adjacent non-malignant renal tissues (See Table S1 in the supplemental material). The PCR products with specific primers for methylated *WNT7A* were detected in 66% (29/44) of the clear cell RCCs analyzed. No DNA methylation was detected in the non-malignant adjacent renal tissues. The PCR products with specific primers for unmethylated WNT7A were detected in all samples analyzed. To check the specificity of the MSP, the PCR products of 7 tumor samples with an identified hypermethylated *WNT7A* gene were sequenced. Data of sequencing confirmed the results of MSPs. Representative MSPs and sequencing of MSP-products are presented in Figure 1A and 1B.

Secondly to verify that MSP determines methylation status of *WNT7A* 5′-CpG-island correctly, bisulfite sequencing was performed for 3 tumor samples that had revealed methylated *WNT7A* 5′-CpG island according to the MSP data. Bisulfite sequencing showed that MSP accurately reflects the methylation status of the *WNT7A* 5′-CpG island in the samples selected (Figure 1C).

To further assess whether hypermethylation of the *WNT7A* 5′-CpG island might be directly responsible for *WNT7A* silencing, the A498 cell line was treated with the DNA methyltransferase inhibitor 5-aza-2′-deoxycytidine. As expected this led to decreased *WNT7A* methylation and restored *WNT7A* expression (Figure 1D).

Hypermethylation of the *WNT7A* gene is significantly higher in tumors at advanced stages (III–IV) than in tumors at early stages (I–II) (p = 0.003). The methylation status of the *WNT7A* gene showed a correlation with the Fuhrman nuclear grade of clear cell RCC: grades (1–2) vs grades (3–4) (p = 0.037). Moreover, *WNT7A* methylation was observed more frequently in patients, older than 50 years (p = 0.012) than in younger patients (Table 2). No correlation was found between the status of *WNT7A* methylation and gender.

**Table 2:** Association of clinical-pathological characteristics and hypermethylation/LOH status of the *WNT7A* gene in clear cell RCCs.

Parameters		Methylated	p*-value	LOH
Age	<50	38% (5/13)	0.012	86% (6/7)
	>50	77% (24/31)		85% (17/20)
Fuhrman nuclear grades 1–2		55%(16/29)	0.037	94% (16/17)
Fuhrman nuclear grades 3–4		87%(13/15)		70% (7/10)
Stages I–II		53%(17/32)	0.003	89% (16/18)
Stages III–IV		100%(12/12)		78% (7/9)

* p-value is referred to correlation between clinical-pathological characteristics and hypermethylation status.

### LOH Analysis of Polymorphic Markers D3S2385, D3S2403 and D3S1252 in Clear Cell RCC

**Figure 2:**
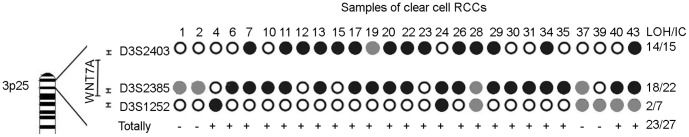
The LOH assays: status of the informative cases of clear cell RCC for the 3q25 region surrounding the *WNT7A* gene. D3S2403, D3S2385, D3S1252– microsatellite markers, white circles - homozygotes (non informative cases), grey circles – absence of the LOH, black circles – presence of the LOH, IC - informative cases, “+” - LOH positive sample, “−” - LOH negative sample.

A copy number study of the chromosome 3p25 region surrounding the *WNT7A* gene was performed using the microsatellite marker analysis. Detection of LOH was performed in 28 samples of clear cell RCCs and the adjacent non-malignant tissues for the polymorphic repeats D3S2403, D3S2385 and D3S1252. The frequency of loss for the above-mentioned markers was 93% (14/15), 82% (18/22) and 29% (2/7) of informative cases, respectively. Among the analyzed samples, 27 cases were informative for at least one LOH marker. Overall, we detected 23 (85%) samples that contained at least one LOH and 4 samples without LOH. In 12 samples one LOH was detected, and in 11 samples - two LOH simultaneously. Data from LOH assays are presented in Figure 2 and Table S1.

### Expression of the *WNT7A* Gene in Clear Cell RCC

**Figure 3:**
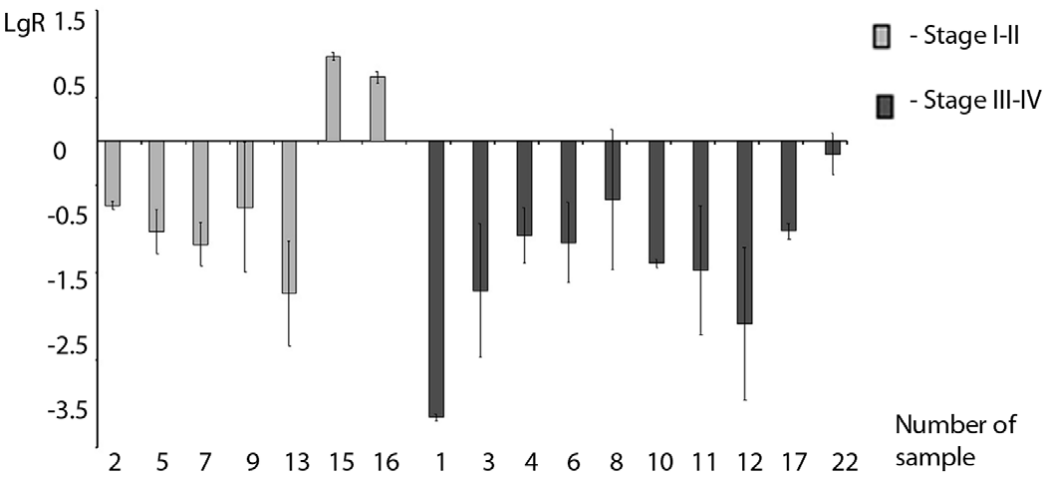
Panel of *WNT7A* gene expression in clear cell RCC samples. R – values of expression in the form of the logarithmic ratio of tumor/normal tissue of the *WNT7A* gene relatively to the *TBP* gene.

We examined whether the expression of the *WNT7A* gene was correlated with DNA methylation/presence of LOH in clear cell RCCs. For this purpose, the expression of the *WNT7A* gene in 17 clear cell renal cell carcinomas was determined by qRT-PCR. A decrease of gene expression was detected in 88% of clear cell RCCs (15/17 samples) (Figure 3 and Table S1). A correlation was detected between the decrease of *WNT7A* gene expression and hypermethylation of the *WNT7A* gene or the presence of LOH (r_s_ = 0.917, p<0.05). The mean values of expression in the form of the logarithmic ratio of tumor/normal tissue for samples with stages I–II (−0.53+/−1.0) and stages III–IV (−1.54+/−0.8) tend to be different (p = 0.09).

### Colony Formation and Proliferation Tests

**Figure 4:**
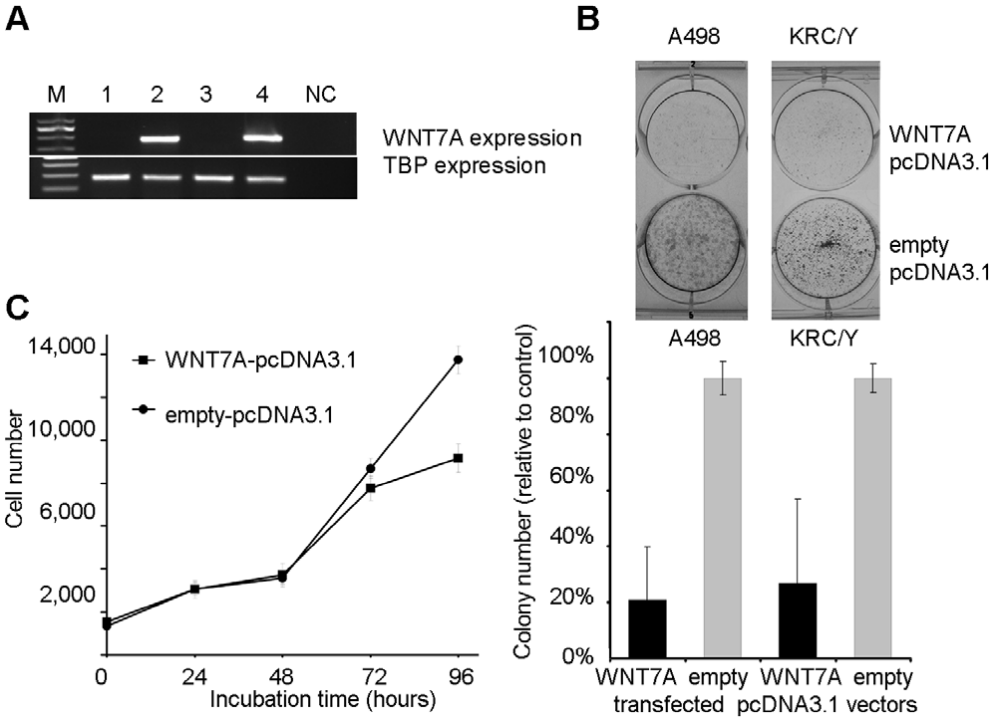
Suppressive effect of *WNT7A* gene re-expression in RCC cell lines. Effect of *WNT7A* gene re-expression (A) on colony formation (B) for the A498, KRC/Y cell lines, and (C) cell proliferation assays for the A498 cell line; M – marker, 1 and 2– A498 cells were transfected by empty-pcDNA3.1 and WNT7A-pcDNA3.1 vectors, 3 and 4– KRC/Y cells were transfected by empty-pcDNA3.1 and WNT7A-pcDNA3.1 vectors, NC – negative control (H_2_0). All experiments were performed in triplicate. Representative results are shown.

The effect of *WNT7A* re-expression on colony formation of the A498 and KRC/Y cell lines was investigated. A high level of the *WNT7A* mRNA was detected in the A498 and KRC/Y cells after transfection with the WNT7A-pcDNA3.1 vector in comparison with the empty vector (Figure 4A). The ectopic expression of the *WNT7A* gene in the A498 and KRC/Y cell lines led to a significant reduction in colony number (p<0.05). The number of A498 WNT7A-pcDNA3.1 and KRC/Y WNT7A-pcDNA3.1 colonies was 20.8% and 26.8% from the number of A498 Empty-pcDNA3.1 and KRC/Y Empty-pcDNA3.1 colonies respectively (Figure 4B).

To further investigate the effect of *WNT7A* on cell proliferation and survival we performed the cell proliferation test for the A498 cell line. There was a significant negative effect of *WNT7A* expression on cell growth in comparison to cells transfected with an empty vector (p<0.05) (Figure 4C).

## Discussion

A large number of tumor suppressor genes are inactivated through DNA hypermethylation of the promoter regions in a wide range of cancers [49]–[51]. Moreover studying of genetic and epigenetic alterations is a powerful tool in searching for novel tumor suppressor genes [52], [53].

To perform a detailed analysis of rearrangements of the *WNT7A* gene in clear cell RCC, the methylation status of the 5′-CpG island of the *WNT7A* gene and the presence of deletions in the locus that corresponds to the *WNT7A* gene were studied. MSP indeed revealed the hypermethylation (66%) of the *WNT7A* gene promoter in clear cell RCC. In comparison, the *WNT7A* gene was hypermethylated in pancreatic carcinomas (71%) [25] and OSCC (78%) [26]. Additionally, it was shown that WNT7A is higher methylated in NSCLC tissue compared to matched normal lung tissues [19], [23], [24]. Thus promoter hypermethylation acts as the main mechanism of the *WNT7A* silencing in a wide range of cancer types.

To investigate the genetic alterations of the WNT7A gene locus, the microsatellite markers analysis of this region on chromosome 3p25 was also performed. The *WNT7A* gene is located between markers D3S2385, D3S2403 and D3S1252. A loss of heterozygosity at least with one marker was found in 85% (23/27) of informative cases. Moreover, for the first time we have shown a loss of the microsatellite markers D3S2385 and D3S2403 in cancer. It should be recalled that the D3S1252 marker was lost in 14% of informative cases of head and neck carcinomas [54].

To verify the correlation between hypermethylation/LOH presence and gene expression, the level of the *WNT7A* gene expression was investigated. Hypermethylation of 5′-CpG island of the *WNT7A* gene/LOH presence coincided with decreased expression of the gene in 88% (15/17) of selected clear cell RCC samples. The methylation/LOH status and expression of *WNT7A* gene have been studied on the same set of samples. Also, we observed that *WNT7A* expression was restored after 5-aza-2′-deoxycytidine treatment of the RCC cell line. In addition, it was reported that expression of the *WNT7A* gene is frequently reduced in lung cancer [55], and that restoration of *WNT7A* gene expression led to growth inhibition of NSCLC cell lines [22]. Importantly, we have found that decreased *WNT7A* expression positively correlates with tumor progression.

A statistically significant correlation exists between the *WNT7A* hypermethylation status and some of the clinical-pathological characteristics. The *WNT7A* gene is more frequently methylated in tumors at advanced stages (III–IV) and high nuclear grades (3–4) than in tumors at early stages (I–II) and low nuclear grades (1–2) of clear cell RCC (Table 2). Similar data were demonstrated in OSCC where methylation of the *WNT7A* gene is characteristic of tumors at advanced stages [26]. At the same time, we did not detect any statistically significant difference of frequency of microsatellite marker loss and any clinical-pathological characteristics.

Based on our data we assume that the *WNT7A* gene could be a potential tumor suppressor gene of clear cell RCC. To support this possibility the tumor suppressor properties of the *WNT7A* gene in RCC cell lines were investigated. For this purpose, the *WNT7A* gene was re-expressed in RCC cell lines A498 and KRC/Y. This led to a significant reduction in colony number in both cell lines. These findings are similar to data obtained previously concerning re-expression of *WNT7A* in NSCLC [21], [22]. In addition, re-expression of *WNT7A* significantly reduced the proliferation rate of the A498 cell line. Thus, the *WNT7A* gene does indeed possess tumor suppressor properties in RCCs.

In summary, genetic and epigenetic alterations play a key role in silencing of the *WNT7A* gene in clear cell RCC. Moreover, restoration of *WNT7A* expression inhibits the growth of RCC cell lines. Therefore, we propose that inactivation of the *WNT7A* gene may play an important role in the development of clear cell RCC.

## Supporting Information

Table S1 **Clinical-pathological characteristics and methylation, LOH, expression status of the WNT7A gene in clear cell RCC samples.**
(DOC)
