# Supplementary material for: Alterations of the WNT7A Gene in Clear Cell Renal Cell Carcinomas
Source: PLoS One. 2012 Oct 8;7(10):e47012. doi: 10.1371/journal.pone.0047012 (PMC3466251; doi:10.1371/journal.pone.0047012)
Supplement: Table S1 — Clinical-pathological characteristics and methylation, LOH, expression status of the WNT7A gene in clear cell RCC samples. (DOC) [file pone.0047012.s001.doc]

Supplementary materials section for

“**Alterations of the *WNT7A* gene in clear cell renal cell carcinomas**”

**Aleksandr G. Kondratov^1§^, Sergiy M. Kvasha^1^, Liubov A. Stoliar^1^, Alina M. Romanenko^2^, Yury M. Zgonnyk^2^, Vasily V. Gordiyuk^1^, Elena V. Kashuba^4,5^, Alla V. Rynditch^3^, Eugene R. Zabarovsky^4,6,7^ and Vladimir I. Kashuba^1^**

1. *Institute of Molecular Biology and Genetics, Department of Molecular Oncogenetics, National Academy of Science, 03680 Kyiv, Ukraine*
2. *Institute of Urology, Academy of Medical Sciences, 04053 Kyiv, Ukraine*
3. *Institute of Molecular Biology and Genetics, Department of Functional Genomics, National Academy of Science, 03680 Kyiv, Ukraine*
4. *Department of Microbiology, Tumor and Cell Biology (MTC), Karolinska Institute, Box 280, Stockholm, S-17177, Sweden*
5. *R.E. Kavetsky Institute of Experimental Pathology, Oncology and Radiobiology, National Academy of Science, Kyiv, 03022 Ukraine*
6. *Laboratory of Structural and Functional Genomics, Engelhardt Institute of Molecular Biology, RAN, 119991 Moscow*
7. *Linkoping University, Faculty of Health Sciences, Department of Clinical and Experimental Medicine, Linkoping, S-58183, Sweden*

**^§^**To whom correspondence should be addressed.

Email: o.g.kondratov@imbg.org.ua

This doc file includes:

Supplementary Table S1

Table S1. Clinical-pathological characteristics and methylation, LOH, expression status of the *WNT7A* gene in clear cell RCC samples.

| **№** | **TNM** | **Sex** | **Age** | **Fuhrman grade** | **DNA methylation status** | **LOH status** | **Expression level** |
| --- | --- | --- | --- | --- | --- | --- | --- |
| 1 | T3N0M0 | M | 65 | 3 | M | negative | DR |
| 2 | T2N0M0 | M | 60 | 4 | M | negative | DR |
| 3 | T3N0M0 | F | 66 | 2 | M | ND | DR |
| 4 | T3N0M0 | F | 62 | 1 | M | positive | DR |
| 5 | T2N0M0 | M | 48 | 3 | M | ND | DR |
| 6 | T3N0M0 | M | 51 | 4 | M | positive | DR |
| 7 | T2N0M0 | M | 68 | 2 | U | positive | DR |
| 8 | T3N0M0 | M | 50 | 3 | M | ND | DR |
| 9 | T2N0M0 | M | 55 | 2 | M | ND | DR |
| 10 | T3N0M0 | F | 60 | 3 | M | positive | DR |
| 11 | T3N0M0 | M | 71 | 3 | M | positive | DR |
| 12 | T3N0M0 | M | 61 | 1 | M | positive | DR |
| 13 | T2N0M0 | F | 58 | 3 | U | positive | DR |
| 14 | T1N0M0 | M | 50 | 2 | U | ND | ND |
| 15 | T2N0M0 | F | 50 | 2 | U | positive | UR |
| 16 | T1N0M0 | F | 22 | 1 | U | ND | UR |
| 17 | T3N1M0 | F | 72 | 2 | M | positive | DR |
| 18 | T2N0M0 | M | 57 | 1 | M | ND | ND |
| 19 | T1N0M0 | M | 57 | 1 | U | positive | ND |
| 20 | T2N0M0 | F | 49 | 1 | U | positive | ND |
| 21 | T3N0M0 | M | 54 | 3 | M | ND | ND |
| 22 | T3N1M0 | M | 58 | 2 | M | positive | DR |
| 23 | T2N0M0 | M | 45 | 4 | U | positive | ND |
| 24 | T2N0M0 | F | 46 | 2 | U | positive | ND |
| 25 | T2N0M0 | F | 52 | 1 | M | NI | ND |
| 26 | T2N0M0 | M | 47 | 1 | U | positive | ND |
| 27 | T2N0M0 | M | 38 | 1 | M | ND | ND |
| 28 | T2N0M0 | F | 46 | 2 | U | positive | ND |
| 29 | T2N0M0 | M | 50 | 2 | M | positive | ND |
| 30 | T2N0M0 | M | 62 | 4 | M | positive | ND |
| 31 | T2N0M0 | M | 64 | 2 | M | positive | ND |
| 32 | T2N0M0 | M | 51 | 2 | M | ND | ND |
| 33 | T2N0M0 | F | 78 | 3 | M | ND | ND |
| 34 | T2N0M0 | M | 38 | 1 | M | positive | ND |
| 35 | T2N0M0 | F | 44 | 2 | M | positive | ND |
| 36 | T2N0M0 | F | 48 | 1 | U | ND | ND |
| 37 | T3N0M2 | M | 53 | 3 | M | negative | ND |
| 38 | T2N0M0 | M | 68 | 2 | M | ND | ND |
| 39 | T2N0M0 | F | 45 | 2 | U | negative | ND |
| 40 | T2N0M0 | M | 69 | 4 | M | positive | ND |
| 41 | T2N0M0 | M | 67 | 4 | M | ND | ND |
| 42 | T2N0M0 | M | 64 | 2 | U | ND | ND |
| 43 | T2N0M0 | M | 66 | 2 | U | positive | ND |
| 44 | T2N0M0 | M | 38 | 2 | M | ND | ND |
